# Nervonic acid as novel therapeutics initiates both neurogenesis and angiogenesis for comprehensive wound repair and healing

**DOI:** 10.3389/fphar.2024.1487183

**Published:** 2024-10-22

**Authors:** Yu-Da Liu, Xiao Peng, Hao-Ran Chen, Xue-Song Liu, Li-Hua Peng

**Affiliations:** ^1^ College of Pharmaceutical Sciences, Zhejiang University, Hangzhou, China; ^2^ Jinhua Institute, Zhejiang University, Jinhua, Zhejiang, China

**Keywords:** nervonic acid, natural product, neural regeneration, angiogenesis, wound repair

## Abstract

Rapid tissue reconstruction in acute and chronic injuries are challengeable, the inefficient repair mainly due to the difficulty in simultaneous promoting the regeneration of peripheral nerves and vascular, which are closely related. Main clinical medication strategy of tissue repair depends on different cytokines to achieve nerves, blood vessels or granulation tissue regeneration, respectively. However, their effect is still limited to single aspect with biorisk exists upon long-time use. Herein, for the first time, we have demonstrated that NA isolated from *Malania oleifera* has potential to simultaneously promote both neurogenesis and angiogenesis *in vitro* and *in vivo*. First, NA was identified by NMR and FTIR structural characterization analysis. In a model of oxidative stress in neural cells induced by hydrogen peroxide, the cells viability of RSC96 and PC12 were protected from oxidative stress injury by NA. Similarly, based on the rat wound healing model, effective blood vessel formation and wound healing can be observed in tissue staining under NA treatment. In addition, according to the identification of nerve and vascular related markers in the wound tissue, the mechanism of NA promoting nerve regeneration lies in the upregulation of the secretion NGF, NF-200 and S100 protein, and NA treatment was also able to up-regulate VEGF and CD31 to directly promote angiogenesis during wound healing. This study provides an important candidate drug molecules for acute or chronic wound healing and nerve vascular synchronous regeneration.

## 1 Introduction

The therapy process of wound includes continuous and complex phases and suffering from a variety of adverse factors, particularly chronic wound such as non-healing diabetic ulcer present one of the most difficult therapeutic challenges in the clinical practice, which is due to excessive oxidative stress, multiple infections, poor neuropathy, and angiopathy (Brazil et al., 2019). Emerging evidence suggests that therapeutic strategies for the promotion of neurogenesis in wound can beneficially accelerate wound healing. However, neurogenesis is not an independent event, and it is tightly mediated by both complicated molecular mechanisms from surrounding neural and non-neural cells. The cellular crosstalk between neural cells and vascular endothelial cells (ECs) plays a key role in neurogenesis (Marrella et al., 2018; Gilbert-Honick and Grayson, 2020). ECs can promote neurogenesis by supporting the high metabolic demands of neural cells, and the bioactive molecules released by the neuroepithelium can also drive the growth and maturation of blood vessels (Marenzana and Arnett, 2013; Chen et al., 2015; Das et al., 2020; Guo et al., 2020; Mi et al., 2021). Both neural cells and ECs constitute a “neurovascular niche” that enhances cell function and accelerates wound healing. Previous studies have confirmed that insufficient vascularization and innervation can lead to delayed tissue regeneration (Fan et al., 2023). Therefore, therapeutics with efficacy in both promoting nerve and angiogenesis might be an important and new key to the rapid tissues and organs regeneration.

However, current tissue regeneration drugs are insufficient in promoting the combination of neurovascular networks to induce vascularization and innervation. In addition to debridement and maintaining a wound environment conducive to healing, various strategies including growth factor and gene delivery as well as cell therapy have been used to enhance the healing of non-healing wounds (Kim et al., 2019). Tissue repair drugs such as vascular endothelial growth factor (VEGF) or nerve growth factor (NGF) delivery approaches in regenerative medicine have a short half-life in the body (Levy et al., 1998), because they are rapidly degraded by proteases and subsequently inactive (Huang et al., 2007), they are quickly eliminated when used alone, and high concentrations can have significant side effects on the human body (Tayalia and Mooney, 2009; Simón-Yarza et al., 2012). As a result, few drugs can specifically promote nerve regeneration and angiogenesis and have significant therapeutic effects on both.

Nervonic acid (NA) has been found to be clinically critical in nerve development and regeneration. It is the fundamental component of the white matter, myelin and cell membranes of nerve fibers, supplementing the glial cell differentiation and myelin regeneration. By combining with sphingoside to form sphingolipid, NA plays the indispensable roles in maintaining the development of nerve cells (Amminger et al., 2012; Kageyama et al., 2018; Lewkowicz et al., 2019; Song et al., 2022) and promoting the repair and regeneration of nerve fibers in damaged tissues (Dhobale et al., 2011; Li et al., 2019). Increasing researches have shown that NA are associated with many neurological diseases such as demyelinating diseases, multiple sclerosis (Lewkowicz et al., 2019; Terluk et al., 2022), Alzheimer’s disease (Astarita et al., 2011), Parkinson’s disease (Amminger et al., 2012; Vozella et al., 2017), cognition (Song et al., 2022), mood symptoms (Kageyama et al., 2018; Kageyama et al., 2021), cardiovascular death (Delgado et al., 2017) and even obesity (Keppley et al., 2020). Various nerve damages cause the NA biosynthesis defects that further induce myelin synthesis disorders, thus leading to abnormal nerve regeneration and impaired axon signal transduction (Love, 2006; Alizadeh et al., 2015; Phung et al., 2023). Accordingly, the administration of external NA has the potential in accelerating the nerve repair and regeneration in wound by promoting the repair of myelin and axons (Gerstl et al., 1972; Ledeen et al., 1973; Nave, 2010; Plemel et al., 2017; Kremer et al., 2019). Although the regeneration of blood vessels is closely related to the recovery of nerve function, direct evidence on the effect of neurotropic drugs such as neuronic acid on angiogenesis is still insufficient. It is hypothesized that neuronic acid could first reconstruct the vascular network by repairing the nerve, and investigated its direct promotion effect on vascular regeneration, confirming its prospect of realizing the promotion of wound repair and healing.

Currently, NA can be purified from plant, while its therapeutic efficacy and application prospect are urgent to be identified. However, the scientific evidence for their effectiveness in wound repair, the peripheral nerve repair and angiogenesis is still very limited.

**FIGURE 1:**
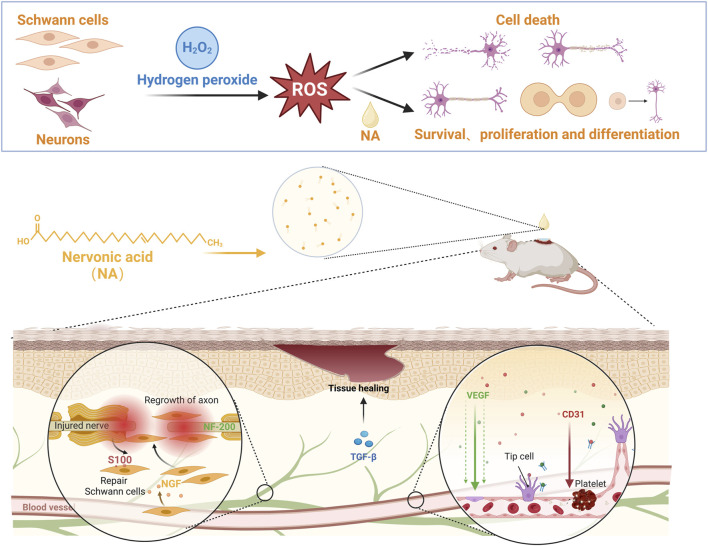
NA accelerate the repair of nerve injury by promoting schwann cells proliferation and nerve growth factor secretion, and stimulate new blood vessels formation by up-regulating the angiogenesis related growth factors levels.

Herein, we have demonstrated for the first time that NA from *Malania oleifera* has potential to simultaneous promote both neurogenesis and angiogenesis *in vitro* and *in vivo* (Figure 1), and location of its physiological mechanism has been investigated. It is expected to provide the scientific evidence for the therapeutic use of NA, as well as identify new effective drug candidate for initiating both nerve and blood vessels regeneration for the complete repair of injured tissues or organs.

## 2 Materials and methods

### 2.1 Materials

NA (cis-15-Tetracosenoic acid, purity ≥ 99%, CAS No: 506-37-6) was obtained from Yunnan Beimu Biotechnology Co., LTD. Major extraction method: the *M. oleifera* kernels are dried, crushed and sieved before being put into a Soxhlet extractor. Then the petroleum ether was added and extracted in a 75°C constant temperature water bath for 7 h. After filtration, the filtrate was distilled. The obtained garlic kernel oil was saponified for 8 h in cold storage, and the pH was adjusted to 2 to obtain the mixed crude solution of neural acid. The concentrated crude neurotic acid was added to absolute ethanol, heated and stirred to fully dissolve, and the purified neurotic acid was obtained by fast dry column chromatography.

Normal saline (0.9%, sterile, CAS No: 7647-14-5) and dimethyl sulfoxide (DMSO, purity ≥ 99.9%, CAS No: 67-68-5) were acquired from Beijing Solarbio Technology Co., Ltd. (Beijing, China). Dulbecco’s modified Eagle’s medium (DMEM), fetal bovine serum (FBS), penicillin, streptomycin, and trypsin were obtained from Gibco BRL (Gaithersburg, MD). H&E staining and Masson’s trichrome staining kit were purchased from Wuhan BiochiDu Biotechnology Co., Ltd. NGF anti-mouse antibody and buffered paraformaldehyde were purchased from Sigma, Inc. (MO, United States). NF-200 (18934-1-AP) and S100 (15146-1-AP) anti-mouse antibody and buffered paraformaldehyde were purchased from Proteintech Group, Inc. (Wuhan, China). CD31 (ab182981) anti-mouse antibody and buffered paraformaldehyde were purchased from Abcam, Inc. (Wuhan, China). VEGF (BA0407) and TGF-β (BA0290) anti-mouse antibody and buffered paraformaldehyde were purchased from Boster, Inc. (Wuhan, China). Horseradish peroxidase (HRP)-labeled (5220-0364) goat anti-mouse antibody and buffered paraformaldehyde were purchased from SeraCare, Inc. (Beijing, China). Hydrogen peroxide solution (500 mL, 3%) was purchased from Sinopharm Chemical Reagent Co., Ltd. and stored at room temperature. All other reagents were of analytical grade.

### 2.2 Methods

#### 2.2.1 Cell culture

Human neuroblastoma cells PC12 (as a neuronal model, were obtained from the Cell Resource Center of Shanghai Institute of Biological Science, Chinese Academy of Science) and rat Schwann cells RSC96 (as a Schwann cell model, were obtained from the Cell Resource Center of Shanghai Institute of Biological Science, Chinese Academy of Science) were cultured in Dulbecco’s modified Eagle’s medium (DMEM). Both cell culture media were supplemented with 10% fetal bovine serum (FBS). Cells were maintained in the cell standard incubator at 37°C in a humidified atmosphere of 5% CO_2_.

#### 2.2.2 Cell viability and proliferation tests

Cell viability was evaluated by using cell counting kit-8 (CCK-8) (AR1160-500, Boster, Wuhan, China) according to the manufacturer’s instructions. About 5,000 cells of PC12 and RSC96 cells were seeded in a 96-well plate per cell and cultured for 24 h. NA of different concentrations (0, 25, 50, 75, 100, 125, 150, 175, 200 μM) were added into each well in pentaplicate. 12 μM and 15 μM of H_2_O_2_ or NA of different concentrations (0, 10, 20, 40, 60, 80, 100, 125, 150, 175, 200 μM) were added into each well in pentaplicate. After incubation for 24 h, the cells were washed by PBS (pH = 7.4) and the culture medium was replaced with 100 μL fresh medium containing 10 μL CCK-8 solution, and incubated at 37°C for 1 h. Finally, the OD value was measured at 450 nm by a microplate reader (Multiskan FC, Thermo Fisher instruments Co., Ltd., United States) and the relative cell viability was presented as percentage of control.

#### 2.2.3 Skin nerve injury in animal model

All animal-related procedures were reviewed and approved by the Animal Advisory Committee at Zhejiang University (ZJU20170733). Sprague–Dawley rats (8 weeks old) were supplied by Zhejiang University Experimental Animal Center, China. 6 rats were included in each investigational group, prior to wound modeling, each rat was assigned a unique identifier, and the hairs on the back of the rats were shaved and removed. The rats were anesthetized by intraperitoneal injection of 30 mg/kg pentobarbital sodium and the dorsal back skin was sterilized by topical application of 10% w/w povidone-iodine solution, followed by washing three times with sterile water. Skin excisions (20 mm × 20 mm) were made by excising the skin within the confines of the square down to the level of subcutaneous panniculus carnosus. Following surgery, rats were monitored during recovery daily to record wound closure. In the experiment, the rat wounds were treated by: 1) 0.2 mL PBS in 10% DMSO, 2) 0.2 mL NA suspension at a concentration of 5 mg/mL in 10% DMSO, 3) 0.2 mL NA suspension at a concentration of 25 mg/mL in 10% DMSO. The rats were given the drug every other day for 18 days and their weight changes were continuously recorded.

#### 2.2.4 Immunofluorescence staining

Paraffin sections (5 μm) were deparaffinized in xylol and then rehydrated in a graded alcohol series. Endogenous peroxidase was inhibited using 3% H_2_O_2_ in methanol for 10 min. The sections were washed with distilled water and then soaked in 0.01 M citrate buffer for epitope retrieval. The sections were washed three times with PBS and incubated with 5% bovine serum albumin at room temperature for 20 min. The samples were subsequently incubated with FITC-conjugated secondary antibodies for 2 h, followed by incubation with DAPI solution (3 μg/mL) for nucleic staining. For immunofluorescence staining, primary antibodies against S100 (1:200, PROTEINTECH GROUP, 15146-1-AP), NF-200 (1:200, PROTEINTECH GROUP, 18934-1-AP), NGF (1:500, Sigma, MO, United States), CD31 (1:1,000, Abcam, ab182981), VEGF (1:200, Boster, BA0407) and TGF-β (1:200, Boster, BA0290), and HRP-labeled anti-rabbit secondary antibodies (1:200, SeraCare, 5220-0364) were used. The cells were observed and photographed under a fluorescence microscope (Olympus BX61) by using Olympus Soft Imaging Solution software (analysis Caseviewer-3DHISTECH Ltd.).

#### 2.2.5 H&E staining

After dewaxing, sections were washed, followed by the addition of hematoxylin (Wuhan BiochiDu Biotechnology Co., Ltd.) for staining for 5 min, hydrochloric acid aqueous solution for 2 s and ammonia aqueous differentiation solution (Wuhan BiochiDu Biotechnology Co., Ltd., B1004) for 15–30 s, and then washed. The slices were dehydrated with 95% alcohol and then stained with eosin solution (Wuhan BiochiDu Biotechnology Co., Ltd.) for 5–8 s. After dehydration and sealing, the slices were observed under microscope.

#### 2.2.6 Masson’s trichrome staining

The reconstituted skin samples, which include full-thickness skin layers, were fixed in 4% paraformaldehyde and processed according to standard procedures for routine light microscopy. The processed tissues were embedded in paraffin. Then, 5 μm sections were stained with Masson’s trichrome stain according to the protocol and were examined and photographed by the Leica image analysis system (Leica, Germany).

#### 2.2.7 Wound healing rate

The wound area was measured on days 0,3, 7, 11, 15 and 19 after the trauma by tracing the wound margin and was calculated using an image analysis program (ImageJ, NIH, MD, United States). The date of complete wound closure was recorded and the wound healing time (WHT) was calculated accordingly.

#### 2.2.8 Structural characterization of NA

The solvent used for NMR analysis is DMSO-*d*_6_ ((CD3)2S = O). A range of 20–25 mg of the sample was dissolved in this solvent and analyzed on a Bruker 600 MHz NMR spectrometer using five different modes: ^1^H experiment, ^13^C experiment with decoupling, ^1^H-^1^H COSY ex-periment, ^1^H-^13^C multiplicity edited HSQC with gradient selection BF1 ≤600 MHz, and ^1^H-^13^C HMBC with gradient selection, and these five modes were scanned and tested. A Nicolet infrared spectrometer (iS 50 FTIR; Nicolet, Glendale, WI, United States) was used to acquire FTIR spectra of NA. A droplet of oil sample was applied onto the Platinum ATR at a temperature of 25°C. Wavenumber ranged from 4,000 to 400 cm^−1^ for 16 scans.

#### 2.2.9 Statistical analysis

Statistical analyses were performed with GraphPad Prism 10.1 (GraphPad Software Inc., CA). All data were reported as the mean ± SEM. Student’s t test was used to compare two groups. Two-way analysis of variance (ANOVA) with Tukey’s multiple comparisons test was performed for multiple comparisons. Value *p* < 0.05 was considered as differences. *p < 0.05, **p < 0.01, ***p < 0.001, ****p < 0.0001. ImageJ software was used for quantification of images.

## 3 Results

### 3.1 The structural characterization of NA from *Malania oleifera*

To identify the NA which were isolated and purified from the *M. oleifera*, ^1^H and ^13^C NMR analysis was first performed. ^1^H-NMR of the NA sample (Supplementary Figure S1) reveals a peak at 11.92 ppm, which corresponds to the carboxyl protons of various fatty acid compounds. The peaks at 5.26 ppm are attributed to the NA. In the spectrum, the signal at 3.42 ppm is from solvent water, while the peak at 2.51 ppm corresponds to protons in the solvent DMSO. The high field range of 0.81–2.13 ppm displays several peaks associated with different saturated alkyl C-H protons of fatty acids. Specifically, the relatively high field signals at 2.13 and 1.94 ppm are attributed to alkyl protons near the carboxyl group, while the -CH_3_ proton signal appears at 0.81 ppm. The majority of remaining alkyl protons are represented by a strong signal at 1.20 ppm ^13^C-NMR of the NA compound (Supplementary Figure S2) reveals a peak at 174.72 ppm, attributed to the carboxyl carbons of NA. Multiple peaks near 129.77 ppm correspond to the olefin carbon signals of NA. The peak at 40.21 ppm represents the carbon signal from DMSO. In the high field range of 14–35 ppm, several peaks correspond to saturated alkyl carbon signals, reflecting the saturated carbons in different long-chain fatty acids. The signals at 34.10 and 31.87 ppm are attributed to alkyl carbons near the carboxyl and olefin groups, while the carbon signal for -CH_3_ appears at 14.16 ppm. A significant number of alkyl carbons are concentrated in the 20–30 ppm range, consistent with the characteristic features of NA ^13^C-NMR. Consequently, the NMR spectra provide reliable structural information confirming the presence of NA.

In addition, the FTIR analysis was performed to obtain structural evidence and information about functional groups and to determine if there was NA. The FTIR spectrum of the combined treatment samples in the region of 4,000-500 cm^-1^ are shown in Supplementary Figure S3. The peaks observed at 2,918 cm^-1^ correspond to functional group hydrogen bond stretching. Specifically, they are indicative of -OH stretching vibrations found in saturated carbon and the symmetric stretching vibrations of the aliphatic CH_2_ groups present in NA. The carbonyl stretching vibration of the carboxylic acid in NA produces a distinct peak at 1,690 cm^-1^. The peak observed at 1,473 cm^-1^ is associated with the -C=C- double bond in NA. The -OH of carboxylic acid has two relatively strong and wide bending vibration absorption peaks at 1,400 cm^-1^ and 936 cm^-1^, which can be used as further evidence to determine the existence of carboxylic acid structure. The peak at 717 cm^-1^ was attributed to the vibration of the disubstituted olefins cis-HC = CH, which overlapped with the vibration of CH_2_. This structural information is similar to molecular data by FTIR, previously reported in other study (Gao et al., 2024). In-dept comparison of these signals indicated that the extracts match the chemical structures present of NA.

### 3.2 The promotion of NA on nerve regeneration

#### 3.2.1 The protection and anti-oxidative stress effects of NA in nerve cells *in vitro*

**FIGURE 2:**
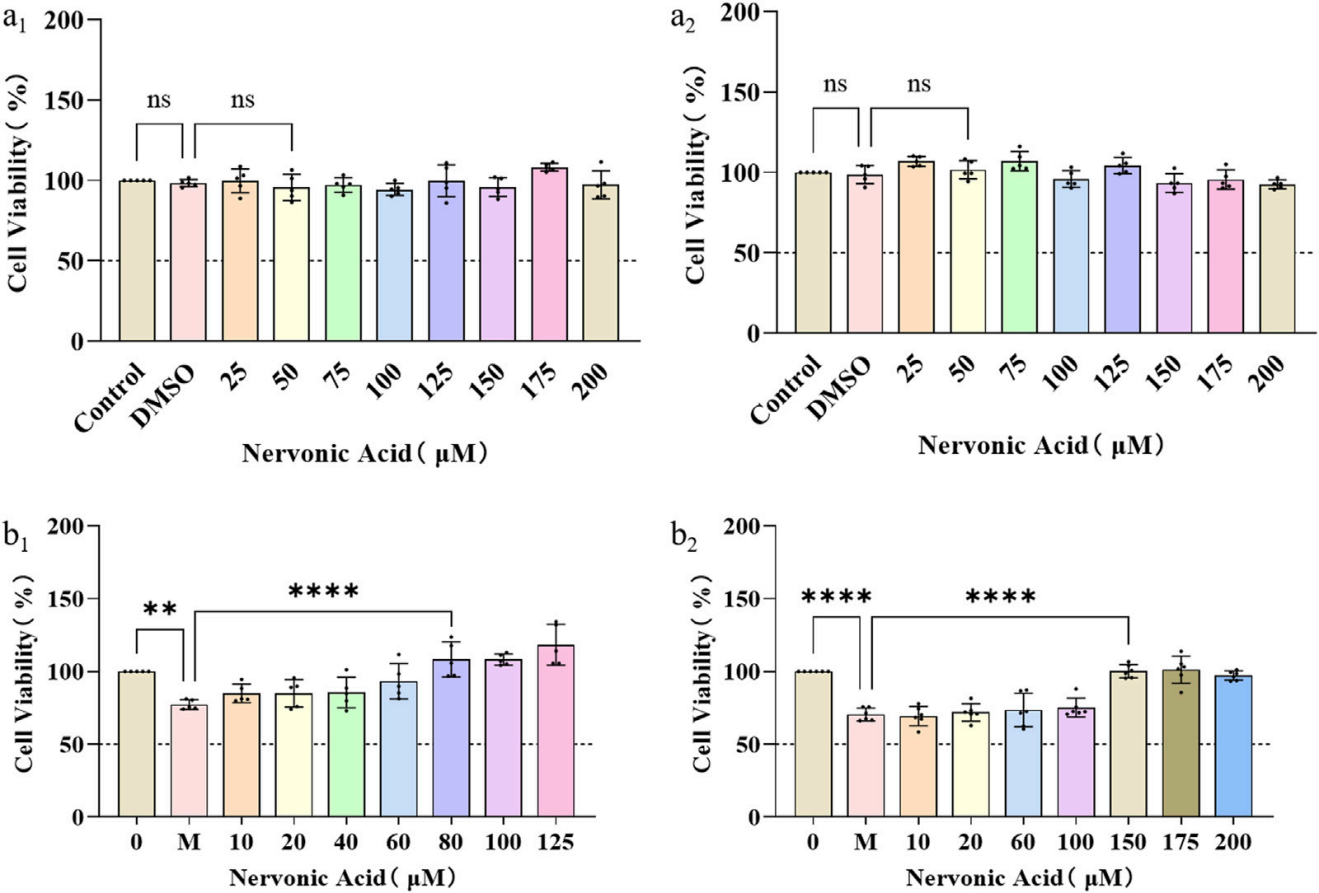
The protection and anti-oxidative stress effects of NA in PC12 and RSC96. **(A_1_,A_2_)** The influence of NA on the cell viability of PC12 (a_1_) and RSC96 (a_2_). **(B_1_,B_2_)** The influence of NA on oxidative stress of PC12 (b_1_) and RSC96 (b_2_) damage caused by hydrogen peroxide. Statistical significance is indicated as ^ns^ *p* ˃ 0.05, **p < 0.01, ***p < 0.001, ****p < 0.0001 versus the DMSO group and the hydrogen peroxide group (M).

SCs are glial cells that have been extensively investigated in the context of nerve repair and are recognized as the typical targets for nerve injury therapy. Maintaining the physiological function of neurons and SCs is particularly important in the process of wound nerve repair. The biocompatibility of NA on nerve cells at different concentrations was first investigated by measuring the cell viability of PC12 and RSC9 under normal culture conditions. It can be seen that NA was not cytotoxic to PC12 (Figure 2A_1_) and RSC96 (Figure 2A_2_) cells at concentrations of 0–200 μM.

Furthermore, in order to study the protective effect of NA on nerve cells and SCs under oxidative stress, the PC12 and RSC96 were treated with H_2_O_2_ at concentration of 0–35 μM for 24 h, respectively, to initially construct a H_2_O_2_-induced injury mode. According to the cell viability by CCK8 method, NA was found to show protective effects on PC12 under 80–125 μM H_2_O_2_ (Figure 2B_1_) and RSC96 at 150–200 μM H_2_O_2_ (Figure 2B_2_), which indicated that NA can avoid the damage of nerve cells from oxidative stress caused by hydrogen peroxide.

#### 3.2.2 The activation and repair effect of NA on nerve cells *in vivo*

**FIGURE 3:**
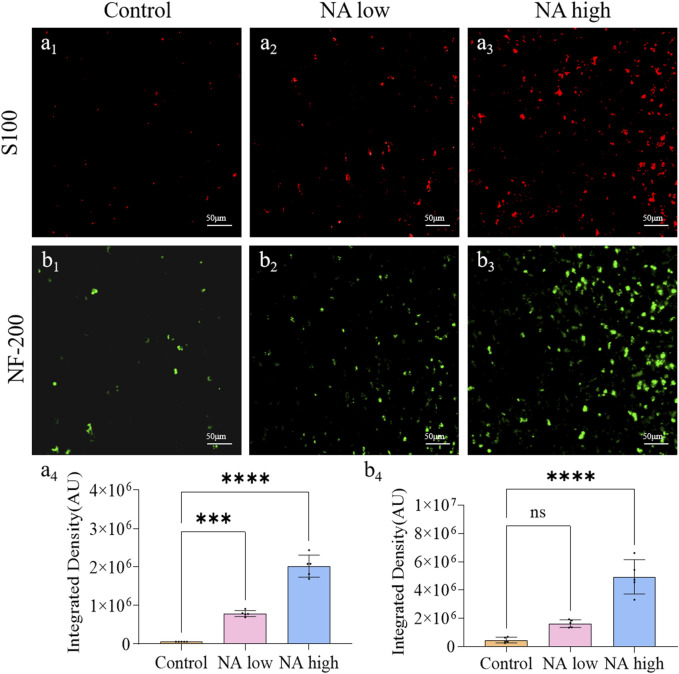
The promotion of NA for the expression of NF-200/S100 *in vivo*. **(A**_**1**_ **–A**_**3**_ **)** Immunofluorescence staining indicating the S100 expression in the healed skins on 11 days post-treatment. **(A**_**4**_ **)** The semi-quantification of S100 *in vivo* on day 11. **(B**_**1**_ **–B**_**3**_ **)** Immunofluorescence staining indicating the NF-200 expression in the healed skins on 11 days post-treatment. **(B**_**4**_ **)** The semi-quantification of NF-200 *in vivo* on day 11. Statistical significance is indicated as ^ns^ *p* ˃ 0.05, ***p < 0.001, ****p < 0.0001 versus the Control group.

Neurofilament protein NF-200 is a cytoskeleton protein of nerve cells, which can completely display the morphology and distribution of the entire nerve cells, including the nucleosome, nerve dendrites and axons of neurons. S100 is a specific protein of the nervous system that reflects the activity and damage of glial cells. Therefore, the expression level of NF-200 and S100 around the wound were characterized to show the degree of Schwann cell activation and the neuronal cell repair effect. On the 11th day, the integrated density of NF-200 (Figures 3B_1_–B_3_) of the NA high group (*p* < 0.001) group (Figure 3B_4_) were significantly higher than the control group. Meanwhile, the integrated density of S100 (Figures 3A_1_–A_3_) of both the NA low group (*p* < 0.001) and NA high group (*p* < 0.0001) group (Figure 3A_4_) were significantly higher than the control group. NA has the ability to promote the activation of SCs in the early stage of wound repair, and promote the repair process of neurons in a certain dose dependent manner.

#### 3.2.3 The promotion of NA for the expression of NGF and TGF-β *in vivo*

**FIGURE 4:**
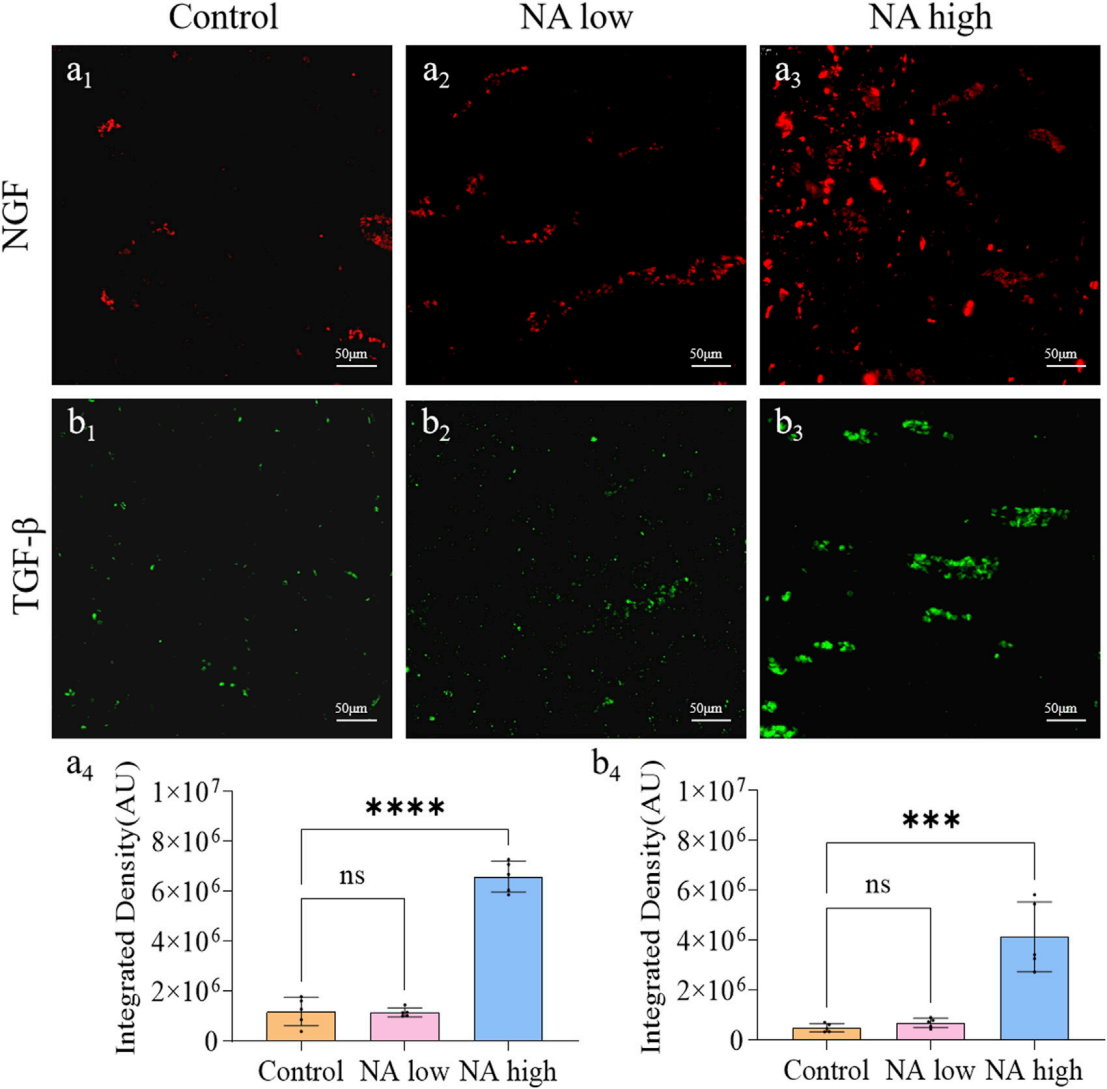
The promotion of NA for the expression of NGF and TGF-β *in vivo*. **(A**_**1**_ **–A**_**3**_ **)** Immunofluorescence staining indicating the NGF expression in the healed skins on 11 days post-treatment. **(A**_**4**_ **)** The semi-quantification of NGF *in vivo* on day 11. **(B**_**1**_ **–B**_**3**_ **)** Immunofluorescence staining indicating the TGF-β expression in the healed skins on 11 days post-treatment. **(B**_**4**_ **)** The semi-quantification of TGF-β *in vivo* on day 11. Statistical significance is indicated as ^ns^ *p* ˃ 0.05, ***p < 0.001, ****p < 0.0001 versus the Control group.

The application of neurotrophic factors was considered to be an effective therapy for the wound peripheral nerve injuries, as they support the regeneration of axons and formation of new myelin sheaths. NGF is the prototype for the neurotrophin family of polypeptides, which are essential for the development and survival of certain sympathetic and sensory neurons in both the central and peripheral nervous systems. The TGF-β was shown to stimulate angiogenesis, fibroblast proliferation, myofibroblast differentiation, and matrix deposition. Experimentally, on the 11th day, the integrated density of NGF (Figure 4A_1_) and TGF-β (Figure 4B_1_) in control group maintained at a low level, which indicate the natural expression of NGF was prevented by the wound. By contrast, both the integrated density of NGF (Figures 4A_3_, A_4_) and TGF-β (Figures 4B_3_, B_4_) in high NA group were significantly higher than the control group. Therefore, NA increased the expression of NGF and TGF-β in the early stage of wound, these results present evidence for the promotion of NA in the accelerated regeneration of neural cells.

### 3.3 The promotion of NA for the expression of CD31 and VEGF *in vivo*

**FIGURE 5:**
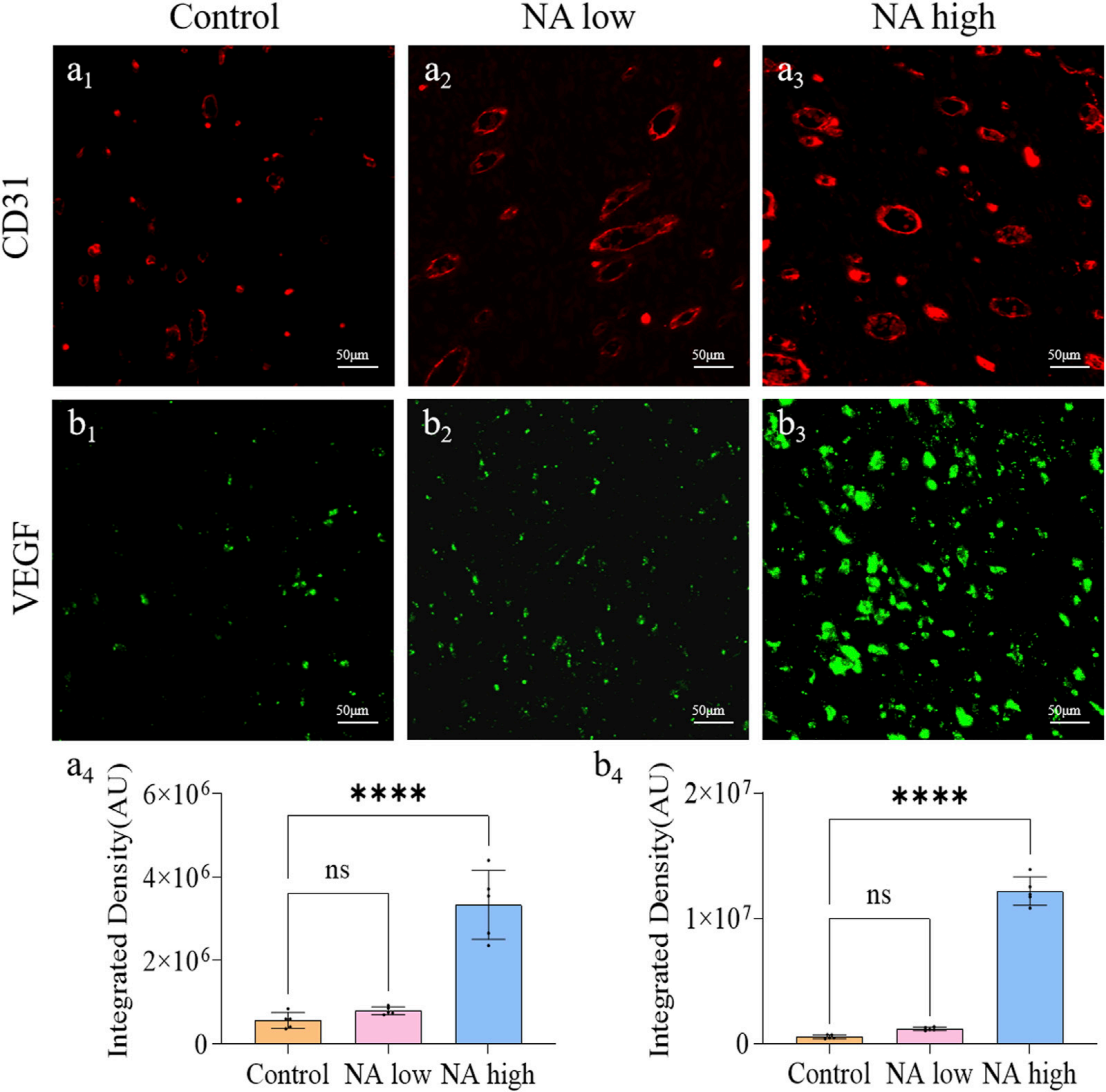
The promotion of NA for the expression of CD31 and VEGF *in vivo*. **(A**_**1**_ **–A**_**3**_ **)** Immunofluorescence staining indicating the CD31 expression in the healed skins on 11 days post-treatment. **(A**_**4**_ **)** The semi-quantification of CD31 *in vivo* on day 11. **(B**_**1**_ **–B**_**3**_ **)** Immunofluorescence staining indicating the VEGF expression in the healed skins on 11 days post-treatment. **(B**_**4**_ **)** The semi-quantification of VEGF *in vivo* on day 11. Statistical significance is indicated as ^ns^ *p* ˃ 0.05, ****p < 0.0001 versus the Control group.

The formation of new capillaries in the original blood vessel network is a key process of wound healing. CD31 is a marker of angiogenesis and can reflect the remodeling of neovascularization, and VEGF has the ability to induce the regeneration of existing blood vessels or the growth of new blood vessels. Therefore, the CD31 and VEGF expression were detected through immunohistochemical staining. As shown in Figure 5, the integrated density of CD31 in NA low group was up-regulated, and the level in NA high group was significantly higher than the NA low and control group (Figures 5A_1_-A_4_). Similarly, the integrated density of VEGF in both NA low group and high group were significantly higher than the control group (Figures 5B_1_-B_4_). There results indicate that NA also has the ability to increase the expression of CD31 and VEGF in wound healing, thereby promoting skin angiogenesis.

### 3.4 The promotion of NA on angiogenesis and tissue repair *in vivo*

**FIGURE 6:**
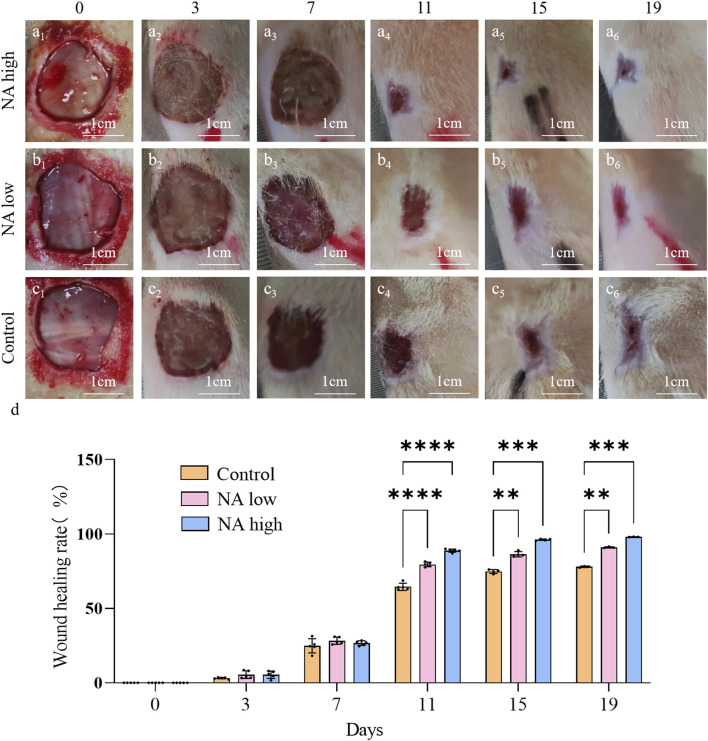
Wound closure under different treatment *in vivo*. **(A**_**1**_ **–A**_**6**_ **)** Representative images of skin wound healing of NA high on 0, 3, 7, 11, 15, 19 days. **(B**_**1**_ **–B**_**6**_ **)** NA low on 0, 3, 7, 11, 15, 19 days. **(C**_**1**_ **–C**_**6**_ **)** Control on 0, 3, 7, 11, 15, 19 days. **(D)** Wound healing rates at different time points of Control, NA low and NA high groups. Statistical significance is indicated as **p < 0.01, ***p < 0.001, ****p < 0.0001 versus the Control group.

No postoperative adverse effects, such as infection, pyogenesis, or body fluid effusion were observed in the animals during the experiment. The wound closure rates on days 0d, 3d, 7d, 11d, 15d and 19d were determined by the percentage of wound surface covered by healed skin and are shown (Figures 6A-D). Briefly, all wounds demonstrated gradual healing, but notably, both the NA low and high group obtained a significantly smaller wound area than the control group from day 11, which indicate that NA has the ability to accelerated the wound closure.

**FIGURE 7:**
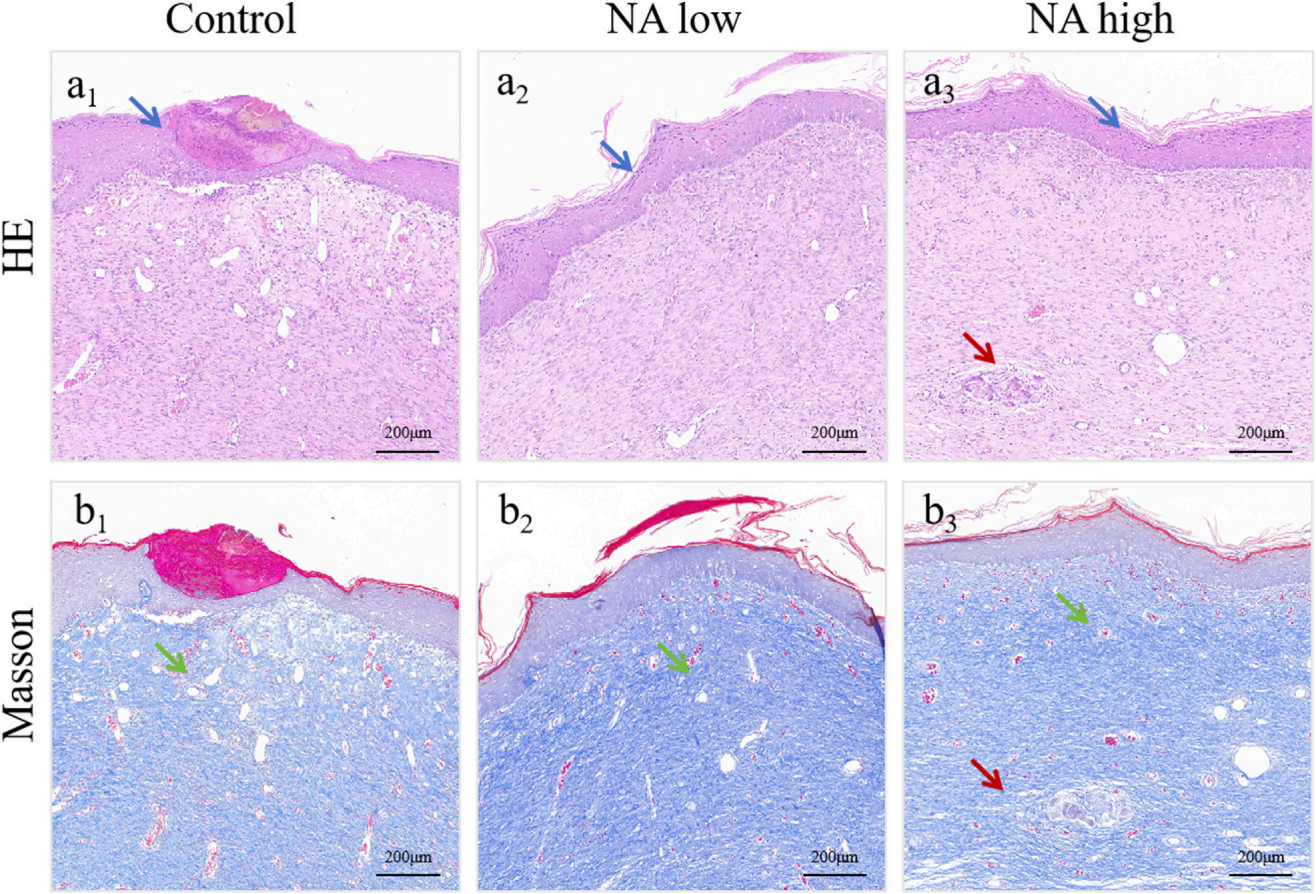
H&E **(A**_**1**_ **–A**_**3**_ **)** and Masson **(B**_**1**_ **–B**_**3**_ **)** staining of healed skin on day 19 post-treatment. The green arrows indicate the blood vessel, the blue arrows indicate the epidermis and the red arrows indicate the skin appendages.

In addition, the wounds and surrounding skin of rats were collected at 11th and 19th days. The results of H&E staining (Figure 7A_1_–A_3_) and Masson staining (Figure 7B_1_–B_3_) for 19d indicated that the distribution of angiogenesis in the skin of rats in NA low group was significantly increased, and the level of angiogenesis was higher than that in the control group. At the same time, angiogenesis increased significantly with the increase of NA dose, and it was found that the NA high group had better cuticle repair than the other two groups.

## 4 Discussion

Acute and chronic wounds undergo multiple stages of healing, especially chronic and refractory wounds such as diabetic ulcers, which are often challenged by excessive oxidative stress, inflammation, and infection, leading to adverse neuropathy and vascular damage, delaying the efficiency of healing. The “neurovascular niche” is a novel theory to guide the treatment of acute and chronic wounds, and the cellular crosstalk between nerve cells and vascular endothelial cells may represent a complex physiological mechanism that needs further study to elucidate. In this study, based on individual nerve cell protection experiments, NA has been proven its unique effect in protecting nerve cells against oxidative stress and maintain nerve cell homeostasis, promoting SCs activation and nerve fiber repair, which is consistent with the reported functions of NA (Guo et al., 2022). The mechanism is closely related to growth factors such as NGF, TGF-β and they are really upregulated owing to the NA treatment. Correspondingly, the expression of neurofilament protein, a marker of nerve fibers, also increased significantly, and there was a high colocalization phenomenon with S100 expression (Qian et al., 2023), indicating that the presence of NA also increased the activity of SCs in the peripheral nervous system, which promotes myelin repair and axon regeneration at skin wounds. At the same time, in the rat wound healing model, angiogenesis was indeed promoted by NA which is regard as a neuro-promoting drug. Based on previous studies that mentioned in section “introduction,” the bioactive molecules released by the neuroepithelium can also drive the growth and maturation of blood vessels, the secretions such as NGF following the reconstruction of nerve function are promising to help angiogenesis. The important role and mechanism of NA in promoting angiogenesis was shown for the first time. It is worth noting that both VEGF and CD31 were found to be significantly increased in parallel with neural-related markers in wounds, suggesting that NA has the potential to directly promote angiogenesis in acute and chronic wounds, and the angiogenesis is not caused by bioactive molecules after nerve reconstruction. Owing to the promotion of NA in constituting “neurovascular niche,” NA also showed a significant promoting effect on the comprehensive wound repair. These results further prove and suggest the great potential of NA in initiating the complete repair of organs and tissues by promoting the regeneration of both blood vessels and nerves, and provides a promising new drug candidate for tissue/organ comprehensive repair and functional reconstruction.

## Data Availability

The original contributions presented in the study are included in the article/Supplementary Material, further inquiries can be directed to the corresponding author.
