# Supplementary material for: Nervonic acid as novel therapeutics initiates both neurogenesis and angiogenesis for comprehensive wound repair and healing
Source: Front Pharmacol. 2024 Oct 22;15:1487183. doi: 10.3389/fphar.2024.1487183 (PMC11534657; doi:10.3389/fphar.2024.1487183)
Supplement: Supplementary file 1 [file DataSheet1.docx]

Supplementary Material

# Supplementary Figures and Tables

## ^1^H and ^13^C spectra of Nervonic acid

**
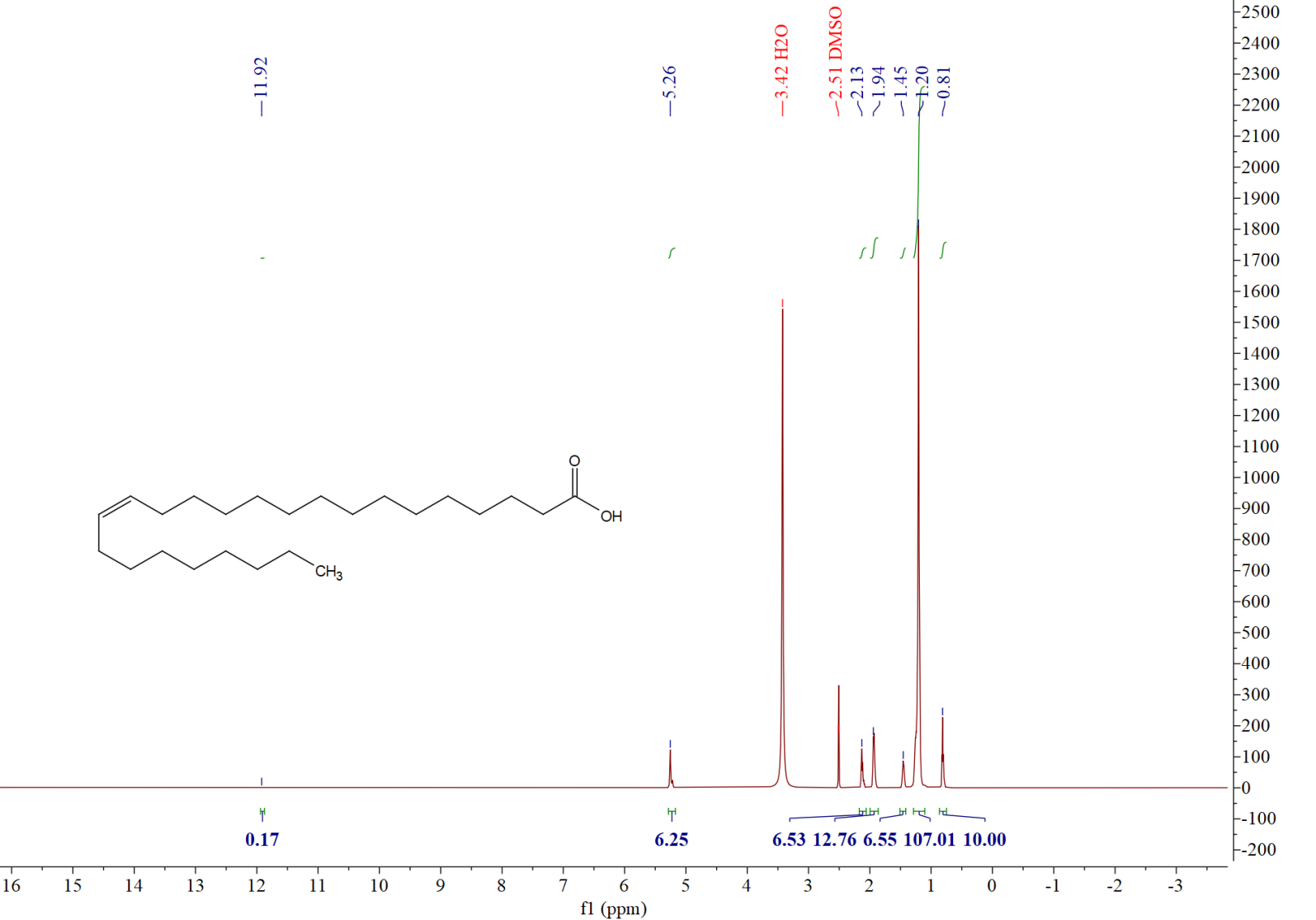
**

**Supplementary Figure 1.** ^1^H NMR (600 MHz, DMSO-*d*_6_) δ 11.92(br,1H), 5.26 (m, 2H), 2.13 (m, 2H), 1.94 (m, 4H), 1.45 (m, 2H), 1.20 (m, 32H), 0.81 (m, 3H).

**
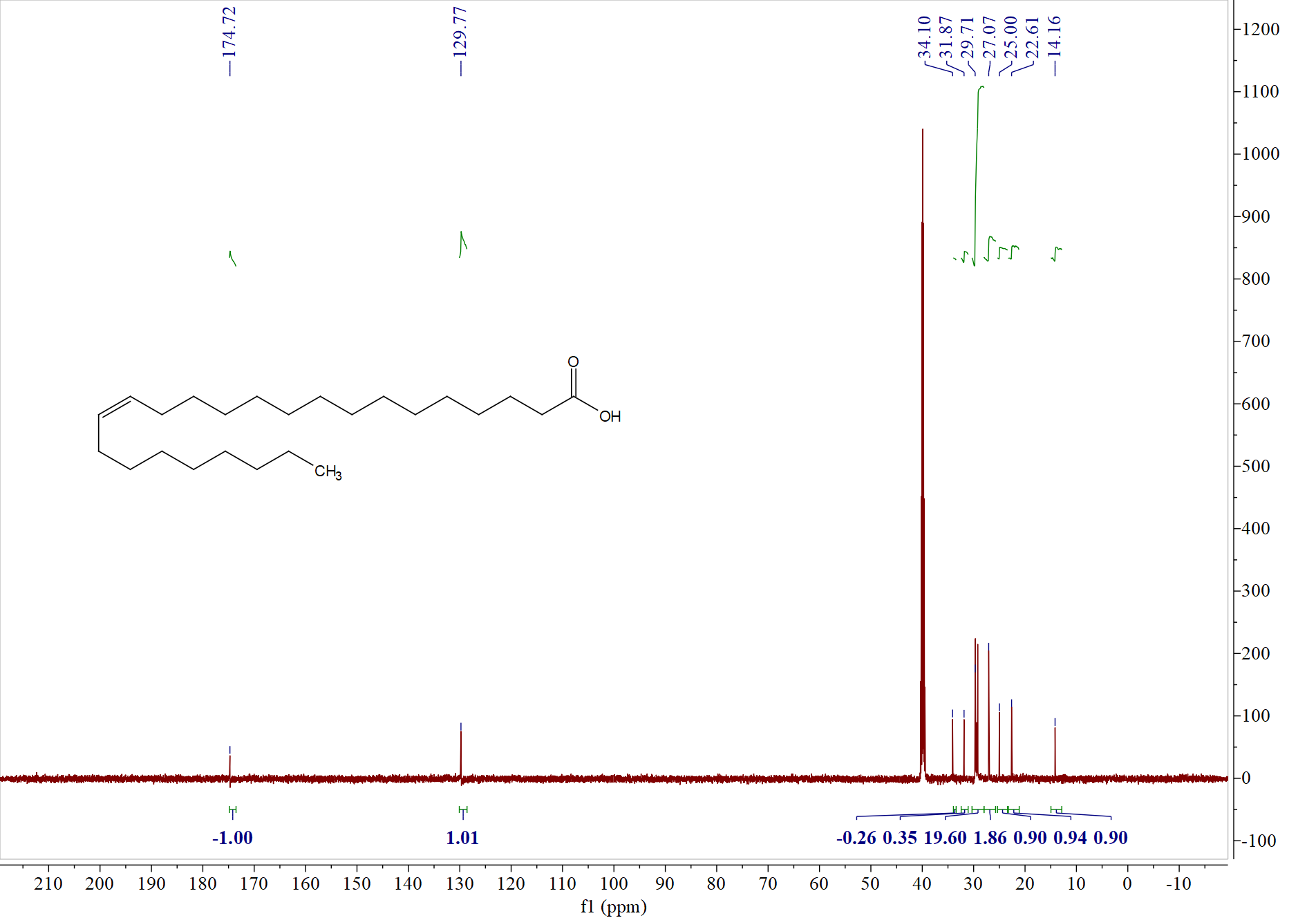
**

**Supplementary Figure 2.** ^13^C NMR (150 MHz, DMSO-*d_6_*) δ 174.72, 129.77, 34.10, 31.87, 29.71, 29.67, 29.61, 29.53, 29.47, 29.43, 29.30, 29.21, 27.07, 25.00, 22.61, 14.16.





**Supplementary Figure 3**. FT-IR analysis of Nervonic acid.
